# Deciphering the role of neddylation in tumor microenvironment modulation: common outcome of multiple signaling pathways

**DOI:** 10.1186/s40364-023-00545-x

**Published:** 2024-01-08

**Authors:** Dequan Liu, Xiangyu Che, Guangzhen Wu

**Affiliations:** Department of Urology, the First Affiliated Hospital of Dalian Medical University, Dalian, 116011 China

**Keywords:** Neddylation, TME, NEDD8, Signaling pathway, MLN4924, Clinical trials

## Abstract

Neddylation is a post-translational modification process, similar to ubiquitination, that controls several biological processes. Notably, it is often aberrantly activated in neoplasms and plays a critical role in the intricate dynamics of the tumor microenvironment (TME). This regulatory influence of neddylation permeates extensively and profoundly within the TME, affecting the behavior of tumor cells, immune cells, angiogenesis, and the extracellular matrix. Usually, neddylation promotes tumor progression towards increased malignancy. In this review, we highlight the latest understanding of the intricate molecular mechanisms that target neddylation to modulate the TME by affecting various signaling pathways. There is emerging evidence that the targeted disruption of the neddylation modification process, specifically the inhibition of cullin-RING ligases (CRLs) functionality, presents a promising avenue for targeted therapy. MLN4924, a small-molecule inhibitor of the neddylation pathway, precisely targets the neural precursor cell-expressed developmentally downregulated protein 8 activating enzyme (NAE). In recent years, significant advancements have been made in the field of neddylation modification therapy, particularly the integration of MLN4924 with chemotherapy or targeted therapy. This combined approach has demonstrated notable success in the treatment of a variety of hematological and solid tumors. Here, we investigated the inhibitory effects of MLN4924 on neddylation and summarized the current therapeutic outcomes of MLN4924 against various tumors. In conclusion, this review provides a comprehensive, up-to-date, and thorough overview of neddylation modifications, and offers insight into the critical importance of this cellular process in tumorigenesis.

## Introduction

 Neddylation is a reversible post-translational modification akin to ubiquitination and is characterized by the reversible covalent conjugation of neural precursor cell-expressed developmentally downregulated protein 8 (NEDD8) with specific substrate proteins [1, 2]. NEDD8, a highly conserved protein in eukaryotes, exhibits predominant nuclear expression and comparatively weak cytoplasmic presence [3]. Initially cloned by Kumar et al. in 1992, NEDD8 shares 60% identity and 80% similarity with ubiquitin, making it the molecule most similar to ubiquitin among all ubiquitin-like proteins [4, 5]. The initial non-Cullin proteins implicated as substrates in NEDD8 research, were associated with Breast Cancer-Associated protein 3 (BCA3), a yeast-derived protein [6]. However, the most extensively studied substrates are cullin-RING ligases, which form the largest family of ubiquitin E3 ligases [7, 8]. CRLs are encoded by more than 600 human genes, of which 518 are protein kinase genes. The ubiquitin-proteasome system facilitates the ubiquitination and degradation of approximately 20% of proteins within cells. This mechanism significantly influences multiple cellular processes and is implicated in various human diseases [9, 10]. In 1998, researchers found that both cullin protein and NEDD8 were highly expressed in various cancer cell types, such as colon cancer and leukemia cells, thereby reinforcing the association of neddylation with cancer progression [11, 12]. In 2009, Soucy T. A. et al. recognized MLN4924 as a potent inhibitor of the NAE, disrupting cullin-RING ligase-mediated protein turnover, and inducing apoptosis in tumor cells [13]. MLN4924 inhibits neddylation by binding to the NAE enzyme, leading to its degradation. This inhibitory action prevents the activation of the NEDD8 protein, thereby obstructing the neddylation process in its entirety. This blockade results in the accumulation of unmodified cullin proteins, which subsequently inhibits the activity of the ubiquitin-proteasome system (UPS). This chain of events culminates in the accumulation of ubiquitinated proteins and triggers DNA damage responses in tumor cells, leading to cell cycle arrest, apoptosis, senescence, autophagy, and alterations in mitochondrial function [14–17].

The etiology of cancer is intrinsically tethered to the intricacies of its TME. This milieu, characterized by the amalgamation of cellular entities such as immune cells, fibroblasts, and endothelial cells embedded within a sophisticated extracellular matrix(ECM), has profound implications for neoplastic evolution [18]. Complex intercellular and matrix-associated interactions within the TME underlie tumor ontogenesis and contribute to the significant challenges of therapeutic refractoriness, such as drug resistance [19]. Historically, conventional therapeutic modalities have been myopic in addressing the protective sanctum that the TME proffers to malignant cells. This review aims to encapsulate and scrutinize the impact of neddylation mechanism on the TME and the anti-tumor effects of MLN4924 based on neddylation. Through this analysis, we sought to provide a comprehensive overview of this significant area of cancer biology.

### Neddylation modification

#### NEDD8 is a key molecule in the neddylation process

NEDD8 shares structural and operational principles with ubiquitin, a regulatory protein involved in diverse cellular functions [20, 21]. Initially identified in mouse brain tissue as a developmentally downregulated gene contributing to its nomenclature, NEDD8 is not exclusive to neural precursor cells or the brain but is ubiquitously expressed across numerous tissues throughout the body, underscoring its essential biological role [22]. Despite its relatively small size (approximately 81 amino acids), human NEDD8 plays a pivotal role in cellular functions via neddylation, a mechanism similar to that of ubiquitination. Neddylation involves the covalent attachment of NEDD8 to target proteins, thereby modulating their function or stability [1, 5]. Although NEDD8 shares around 60% of sequence identity with ubiquitin and has a similar three-dimensional structure composed of a beta-grasp fold, the two are not interchangeable. They conjugate to different protein subsets and regulate distinct aspects of cellular biology [23, 24]. Thus, NEDD8, despite its small size, serves as a potent regulatory protein that functions akin to ubiquitin through neddylation. Further understanding its function would provide promising novel insights into cell biology and potential therapeutic strategies, given its involvement in various cellular processes.

#### The cullin family

Proteins belonging to the cullin family serve as integral structural elements of CRLs. These modular E3 ubiquitin ligase complexes play an instrumental role in protein ubiquitination and degradation mediated by the 26 S proteasome [25, 26]. In mammals, this family encompasses proteins such as cullin-1, cullin-2, cullin-3, cullin-4 A, cullin-4B, cullin-5, cullin-7, and the p53-associated parkin-like cytoplasmic protein (PARC). Despite the structural similarity and shared conservation of the Cullin homology domains, these proteins participate in diverse complexes and target unique sets of substrates for degradation. Cullin1, for instance, forms the Skp1–Cul1–F-box (SCF) E3 ubiquitin ligase complex that targets proteins for degradation, including cell cycle regulators. It is also significantly involved in DNA damage response and repair processes [27]. Cullin2 and cullin5, on the other hand, form E3 ubiquitin ligase complexes with Elongin BC and SOCS box proteins, contributing to the hypoxia response and innate immunity [28, 29]. Similarly, cullin3 forms E3 ligase complexes with BTB domain proteins, regulating the oxidative stress response, neuronal morphology, and cardiovascular development [30]. Cullin-4 A and cullin4B are integral in forming E3 ligase complexes with DNA damage-binding protein 1 (DDB1) and DDB1- and CUL4-Associated Factor (DCAF) proteins. They play a role in DNA repair, replication, and cell cycle control, and cullin4A is involved in neddylation in the Hippo pathway [31, 32]. Cullin7, forming an E3 ligase complex with S-phase kinase-associated protein 1 (SKP1), F-box and WD repeat domain-containing 8 (FBXW8), and Regulator of cullins-1 (ROC1), is associated with growth regulation, embryonic development, and glucose metabolism [33]. Lastly, cullin-9, also known as PARC, is involved in the negative regulation of p53 and plays a crucial role in cellular responses to DNA damage and stress [34]. Thus, the diverse functions of Cullin proteins underscore their crucial roles in maintaining cellular homeostasis and response to various stimuli. These complexes are integral to regulating several cellular processes, encompassing cell cycle progression, DNA damage response, signal transduction, and development. This underscores the indispensable role of cullins in upholding cellular homeostasis [35, 36].

Cullin proteins, characterized by their elongated forms, serve as scaffolds that facilitate the assembly of other CRL components. The C-terminus of cullins binds to a RING-finger protein, typically either ring box protein 1 (RBX1)/ROC1 or ring box protein 2 (RBX2)/ROC2/ sensitive to apoptosis gene (SAG), facilitating the recruitment of an E2 ubiquitin-conjugating enzyme [7]. RBX1, also recognized as ROC1, is an essential component of CRL complexes, where it acts as a scaffold protein aiding the transfer of ubiquitin from E2 to the target protein [7]. Conversely, RBX2, also identified as ROC2 or SAG, mirrors the function of RBX1 but exhibits more restricted expression. Nonetheless, they play a unique role in safeguarding cells against apoptosis and fostering cell growth and survival, particularly in of cancer [37]. The N-terminus of cullins interacts with various adaptor proteins and substrate receptors, thereby dictating the substrate specificity of each CRL [7]. The regulatory mechanism of cullin proteins involves neddylation, a process in which the NEDD8, modifies cullins. The conjugation of NEDD8 inductes a conformational shift in the cullin protein, bringing the E2 enzyme close to the substrate thereby enhancing ubiquitin transfer efficiency [36, 38]. Given their pivotal roles in protein degradation, aberrations in cullin function or neddylation have been implicated in various pathological conditions, particularly cancer. This insight has led to the development of pharmaceutical inhibitors targeting the neddylation pathway as potential therapeutic interventions for cancer [13].

#### Neddylation modification process

**Fig. 1:**
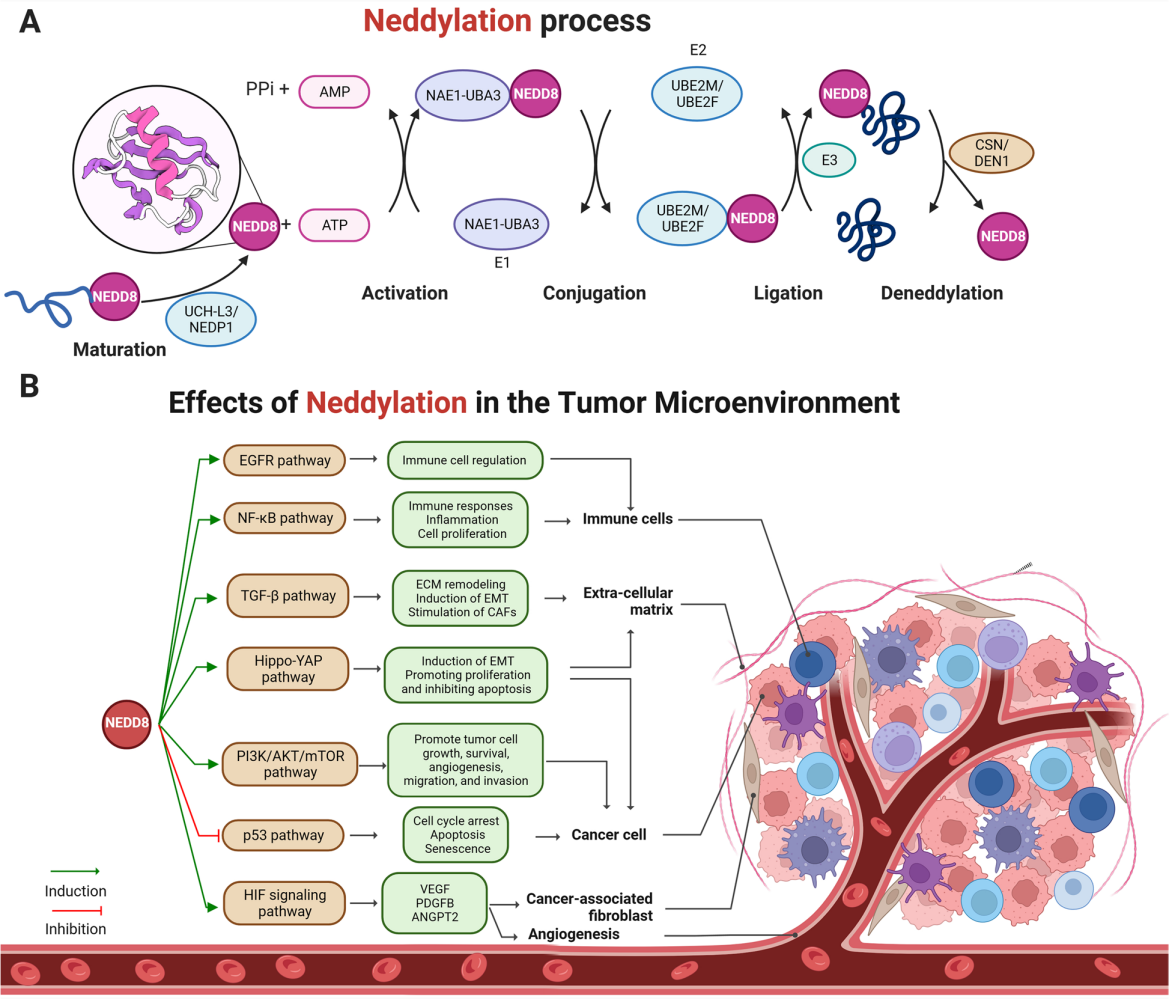
Neddylation, a complex multi-step process involving the post-translational attachment of the NEDD8 protein to target proteins, carries out various cellular functions and protein degradation. This process involves the maturation, activation, conjugation, ligation, and deneddylation stages and is conducted by specialized enzymes such as NEDP1, UBA3, and UBE2M. On the other hand, the neddylation modification significantly influences on the tumor microenvironment. This modification can impact various factors, including VEGF, PDGFB, ANGPT2, the EMT process, CAFs, and the ECM. Together, these two figures highlight the critical role of neddylation in both general cell function and the specific context of tumor progression. NEDD8, neural precursor cell expressed developmentally downregulated protein 8; UCH-L3, ubiquitin C-terminal hydrolase-L3; NEDP1, NEDD8 Protease 1; NAE1, NEDD8-activating enzyme 1; UBA3, ubiquitin-like modifier activating enzyme 3; UBE2 M/F, ubiquitin conjugating enzyme E2 M/F, CSN, COP9 signalosome; VEGF, vascular endothelial growth factor; PDGFB, platelet-derived growth factor B; ANGPT2, angiopoietin 2; EMT, epithelial-to-mesenchymal transition; CAFs, cancer-associated fibroblasts; ECM, extracellular matrix. Created with BioRender.com

Neddylation covalently binds the NEDD8 to specific lysine residues of target proteins post-translationally [39]. This process is similar to ubiquitination and involves the attachment of ubiquitin to target proteins to regulate their stability and function. The overall neddylation process is as follows (Fig. 1A):

- Maturation: Before incorporation into the neddylation pathway, NEDD8 undergoes maturation. Initially synthesized as a precursor protein, NEDD8 contains additional amino acids following the C-terminal diglycine motif, which must be removed to expose this critical motif for subsequent conjugation steps. This maturation is achieved by specific proteases, notably NEDD8 Protease 1 (NEDP1/SENP8) and ubiquitin C-terminal hydrolase-L3 (UCH-L3), which cleave the precursor to yield the mature form of NEDD8. NEDP1, a cysteine protease, recognizes and binds to the precursor NEDD8 and cleaves the additional amino acids by catalyzing the hydrolysis of the peptide bond between the C-terminal glycine (Gly76) of the diglycine motif and the adjacent amino acid, thus exposing the C-terminal diglycine motif [40, 41]. UCH-L3, a member of the UCH family of deubiquitinating enzymes, is also involved in this process. Despite its primary role in the processing and recycling of ubiquitin or ubiquitin-like proteins, UCH-L3 also cleaves the precursor form of NEDD8 [42].
- Activation: This process begins with the activation of NEDD8. The E1 activating enzyme for NEDD8 is a heterodimer of amyloid β precursor protein-binding protein 1, also known as NAE1 and ubiquitin-like modifier activating enzyme 3 (UBA3) [43, 44]. The NAE heterodimer binds to and activates NEDD8 in an ATP-dependent manner, consuming ATP to adenylate NEDD8’s C-terminal glycine and forming a thioester bond with UBA3’s catalytic cysteine [45].
- Conjugation: Following activation, NEDD8 is converted to an E2 conjugating enzyme. There are two known E2 enzymes involved in neddylation: UBE2M, the ubiquitin-conjugating enzyme E2 M (also known as UBC12), and UBE2F, the ubiquitin-conjugating enzyme E2 F. The E2 enzyme carries activated NEDD8 to the E3 ligases [42]. The conjugation of NEDD8 to UBE2M or UBE2F involves a trans-thioesterification reaction that transfers NEDD8 from UBA3 to E2, creating a thioester linkage between the C-terminal glycine of NEDD8 and E2 [46, 47].
- Ligation: The final step involves the transfer of NEDD8 from the E2 enzyme to the target protein, mediated by an E3 ligase. The best-characterized E3 ligase for neddylation is the CRL family, which consists of a cullin protein, a RING domain protein (RBX1 or RBX2), and a substrate recognition component. E3 ligase facilitates the formation of an isopeptide bond between the C-terminal glycine of NEDD8 and a lysine residue on the target protein. The precise lysine residue that is neddylated can varies depending on the specific substrate and context [9, 48].
- Deneddylation: Deneddylation, the reverse of neddylation, involves the removal of NEDD8 from its conjugated proteins, which plays a crucial role in regulating protein function and cellular processes. Deneddylases are specific enzymes involved in this process. The NEDD8-specific protease NEDP1 (also known as DEN1 or SENP8), a primary enzyme involved in deneddylation, recognizes and binds to NEDD8-conjugated proteins and cleaves the isopeptide bond between NEDD8 and the substrate protein, thereby removing the NEDD8 moiety [41, 49]. Notably, deneddylation was not conducted solely by NEDP1. The COP9 signalosome (CSN), a multi-subunit protein complex, also exhibits deneddylase activity, primarily deneddylating the cullin subunits of CRL complexes, a key regulatory event in CRL activity [50]. This activity depends on the JAMM (JAB1/MPN/Mov34 metalloenzyme) motif located in the CSN5(COP9 Signalosome Subunit 5) subunit, which coordinates the necessary zinc ion for catalysis [50].

Neddylation can profoundly influence various aspects of a protein’s function, such as stability, localization, and activity [51]. The critical nature of neddylation makes it a tightly regulated process, ensuring a balanced and coordinated response to cellular demands [51]. However, the dysregulation of neddylation has serious implications, with increasing evidence pointing to its role in disease pathogenesis. Recently, we found that alterations in the neddylation process are present in the TME, where abnormal neddylation can drive uncontrolled cell growth and resistance to apoptosis [52]. This underlines the importance of further studies on the regulatory mechanisms of neddylation and its therapeutic potential in the TME (Fig. 1B).

### Neddylation regulates the TME through various signaling pathways: comprehensive frontier information update

The TME is a complex milieu that encapsulates tumor cells and is defined by intricate interactions between neoplastic cells and the various TME constituents [53]. Recent studies have highlighted neddylation’s pivotal role in shaping the TME, influencing malignant cells, immune cells, vascular networks, the epithelial-to-mesenchymal transition (EMT) and the ECM [54, 55]. Neddylation regulation within the TME has profound implications for tumor prognosis [56].

#### Regulation of tumor cells in TME by neddylation

Tumor cells reshape the TME to enhance cancer progression and resist therapies. They drive angiogenesis via angiogenic factors such as vascular endothelial growth factor (VEGF) [57], produce immunosuppressive factors, and amplify immunosuppressive cells such as myeloid-derived suppressor cells and regulatory T cells (Tregs) to evade the immune response [58]. Tumor cells also lay the groundwork for metastasis by secreting factors that condition distant tissues, establishing a favorable “pre-metastatic niche” for disseminated tumor cells to survive and proliferate [59]. Through metabolic reprogramming, these cells acidify the TME, promote tumor growth and therapy resistance, and impair immune function [60]. Research has shown that activating CRLs by neddylation of cullin proteins promotes cancer cell proliferation by regulating the cell cycle [5]. Furthermore, neddylation modulates apoptosis, affects the DNA damage response by influencing repair protein activity, and shapes the TME by directing cytokine and growth factor secretion [61, 62]. Given its extensive influence on tumor progression and treatment response, targeted neddylation has become a promising anti-cancer therapeutic strategy.

#### Regulatory mechanisms of neddylation in tumor cells: insights from the p53 and phosphoinositide 3-kinase (PI3K)/AKT/mechanistic Target of Rapamycin (mTOR) pathways

**Fig. 2:**
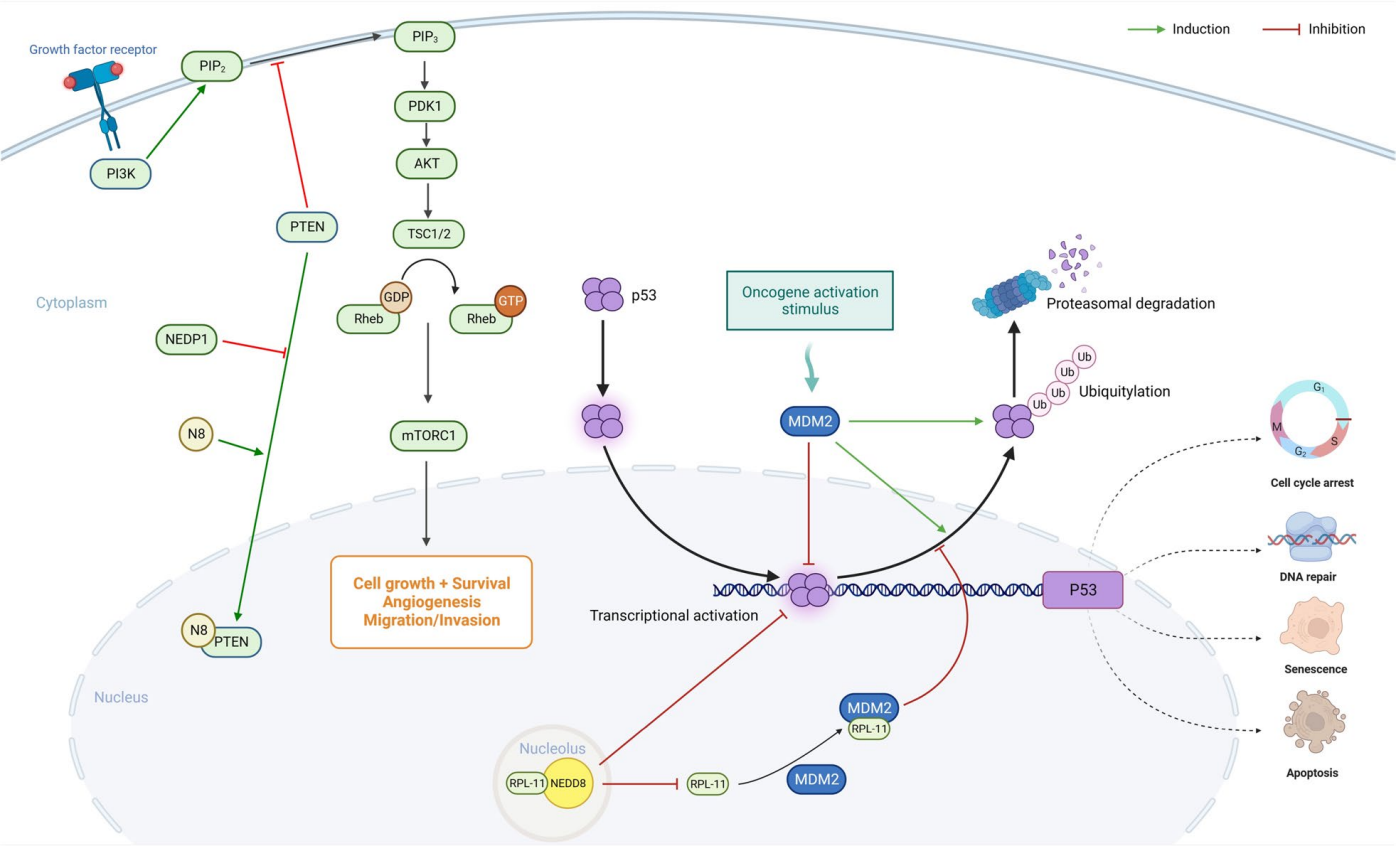
The interplay between the RPL11-MDM2-p53 and PI3K/AKT/mTOR pathways can be regulated by neddylation. The binding of RPL11 to MDM2 inhibits MDM2’s E3 ligase activity, preventing p53 degradation, but neddylation can hinder this binding, indirectly causing p53 degradation and affecting the expression of various target genes. On the other hand, the PI3K/AKT/mTOR pathway activation initiates when growth factors or hormones bind to cell surface receptors like RTKs or GPCRs, leading to the recruitment and activation of PI3K, which then turns PIP2 into PIP3. PIP3 acts as a docking site for proteins such as AKT and PDK1, allowing PDK1 to activate AKT. Activated AKT inhibits the TSC, a negative regulator of mTORC1, thus enabling Rheb to activate mTORC1. This pathway can be negatively regulated by PTEN, which dephosphorylates PIP3 back to PIP2, removing AKT’s activation signal. Neddylation can enhance PTEN’s nuclear translocation, strengthening the pathway’s signal transduction, while deneddylation of PTEN, dependent on NEDP1, can inhibit the PI3K/AKT/mTOR pathway. MDM2, mouse double minute 2 homolog; RPL11, ribosomal Protein L11; Ub, ubiquitin; NEDD8, neural precursor cell expressed developmentally downregulated protein 8; RTKs, receptor tyrosine kinases; GPCRs, G protein-coupled receptors; PI3K, phosphoinositide 3-kinases; PIP2, Phosphatidylinositol 4,5-bisphosphate; PIP3, Phosphatidylinositol 3,4,5-trisphosphate; TSC, tuberous sclerosis complex; mTORC1, mTOR complex 1; AKT, AKT serine/threonine kinase; PDK1, 3-Phosphoinositide Dependent Protein Kinase-1; Rheb, Ras homolog enriched in brain. Created with BioRender.com

The p53 pathway is an important mechanism by which neddylation regulates cancer cells. In general, the p53 pathway is central to regulating cancer cells in response to DNA damage. Depending on the severity of the damage, p53 either halts the cell cycle for repair or induces apoptosis, preventing unchecked cell growth [63–66]. However, ribosomal protein L11 (RPL11), typically involved in protein synthesis, impacts the p53 pathway [67, 68]. When ribosome biogenesis is perturbed, RPL11 binds to Mouse double minute 2 homolog (MDM2), an E3 ubiquitin ligase that targets p53, thus preserving p53 by preventing its degradation [69, 70]. Notably, neddylation intervenes in this pathway by inhibiting the nucleolar release of RPL11, shielding it from degradation. This intervention obstructs the formation of the RPL11-MDM2 complex, which would otherwise inhibit p53 and indirectly cause p53 degradation [71, 72]. This mechanism can effectively shift the p53 pathway towards malignancy (Fig. 2).

Neddylation has a similar regulatory effect on neoplastic cell behavior via the PI3K/AKT/mTOR signaling pathway. Initiated by the binding of RTKs or GPCRs [73], this pathway activates PI3K which converts PIP2 to PIP3 [74]. This conversion enables AKT kinase activation via PDK1 [75]. Activated AKT inhibits TSC, boosting mTORC1 activity, which is a pathological hallmark of various conditions, and allows Rheb to activate mTORC1 [76]. However, this pathway is modulated by PTEN, which reverts PIP3 to PIP2, thereby negating AKT’s activation signal [77]. Recent studies have shown that PTEN undergoes neddylation, potentially facilitating its translocation to the nucleus [78]. This modulation subsequently amplifies the signal transduction within the PI3K/AKT/mTOR pathway [78]. Notably the deneddylation of PTEN, mediated by NEDP1, can attenuate the signal propagation in the PI3K/AKT/mTOR pathway [78] (Fig. 2).

#### Regulation of infiltrated immune cells in the TME by neddylation

Neddylation within the TME critically modulates immune cell functions, impacting tumor-associated macrophages (TAMs), T-cells, B-cells, and dendritic cells, primarily through the nuclear factor kappa light chain enhancer of activated B cells (NF-κB) and epidermal growth factor receptor (EGFR) pathways. This underscores its potential as a therapeutic target in cancer.

#### Regulation of the TAMs by neddylation

TAMs are the predominant leukocytes within tumors and are derived from circulating monocytes drawn to the tumor by chemotactic signals. Once in the TME, these cells differentiate and often adopt M2-like phenotypes. This phenotype is modulated by specific local environmental factors and plays a pivotal role in facilitating tumor progression. Studies have shown that neddylation mediates the production of pro-inflammatory cytokines by macrophages. Multiple studies have pointed out that inhibiting the neddylation process can inhibit lipopolysaccharide (LPS)-induced inflammatory cytokine production, such as interleukin (IL)-6, tumor necrosis factor-alpha (TNF-α), and IL-1β [79, 80]. Additionally, inactivating neddylation curtails inflammation by disrupting lipid metabolism via cullin-6-mediated inhibitor of κB (IκB) degradation, blocking NF-κB activation, which not only modulates macrophage function but also influences cell cycle, apoptosis, and macrophage survival [81, 82]. In conclusion, targeting the neddylation pathway in macrophages, owing to its significant role in tumor progression, offers a promising cancer therapeutic strategy.

#### Regulation of T-cells by neddylation

T-cells are actively triggered to kill cancer cells when their receptors identify unique malignancy-specific antigens. However, the TME can hinder T-cell function using inhibitory molecules such as programmed death-ligand 1 (PD-L1) or Tregs [83, 84]. This underscores the critical role of T-cell modulation in the trajectory of tumor progression. The neddylation pathway governs T-cell function via several mechanisms, with research indicating that its inhibition hampers T-cell receptor/CD28-driven proliferation and cytokine production both in vitro and in vivo, concurrent with diminished extracellular signal-regulated kinase (ERK) activation, underscoring the regulatory involvement of ERK [85]. Pharmacological and genetic manipulations of the neddylation pathway have been shown to modulate T-cell mediated immune responses [86]. Additionally, emerging evidence suggests that modulation of the neddylation pathway, such as MLN4924 treatment, influences T-cell growth, cytokine production, and differentiation, emphasizing its significant role in T-cell function [87, 88]. In summary, the neddylation pathway plays a crucial role in T-cell functionality and modulation, affecting their response to tumor antigens and overall tumor progression. This serves as a direction for future research exploring the regulatory implications of neddylation on T-cell activity.

#### Regulation of B-cells by neddylation

As an anti-neoplastic countermeasure, B-cells operationalize a cascade of antigen-specific antibody synthesis and meticulous antigenic presentation to T-lymphocytes. However, -regulatory B-cells contravene this immune propitiousness by promoting the action of immunosuppressive cytokine IL-10, thereby influencing tumor progression and shaping therapeutic outcomes [89]. Studies have suggested that using MLN4924 to inhibit CRLs results in accumulating CRL substrates like IκB and in a CD40L-expressing stromal co-culture system. Both continuous and pulse treatments with MLN4924 suppress NF-κB activity in CLL B-cells ex vivo, and alkylating agents bendamustine and chlorambucil amplify MLN4924-induced DNA damage and apoptosis thereby improving therapeutic efficacy [90–92]. In conclusion, the aforementioned research underscores the promising potential of MLN4924 for augmenting the therapeutic effectiveness of B-cell targeted interventions.

#### Regulation of dendritic cells (DCs) by neddylation

DCs serve as sentinels for presenting tumor-derived antigens to T-cells [93, 94]. However, the TME can impair their maturation and function by producing immunosuppressive molecules such as IL-10 and VEGF [95]. Dysregulated neddylation leads to aberrant DC responses and is implicated in the pathogenesis of multiple malignancies [86]. Neddylation targeting impedes DC maturation by reducing cytokine production and down-regulating costimulatory molecules while promoting caspase-dependent apoptosis, a process linked to mTOR inactivation due to cullin-1 deneddylation-induced deptor accumulation [96]. By inhibiting neddylation, there is a marked reduction in proinflammatory cytokine release from DCs, outperforming the effects of bortezomib or dexamethasone, and a diminished capacity of DCs to activate murine and human allogeneic T cells, positioning neddylation blockade as a promising approach for modulating immune-mediated diseases [97]. In conclusion, targeting neddylation in dendritic cells offers a potential therapeutic strategy for modulating immune responses in various malignancies.

#### Regulatory mechanisms of neddylation in infiltrated immune cells: insights from the NF-κB and EGFR pathways

**Fig. 3:**
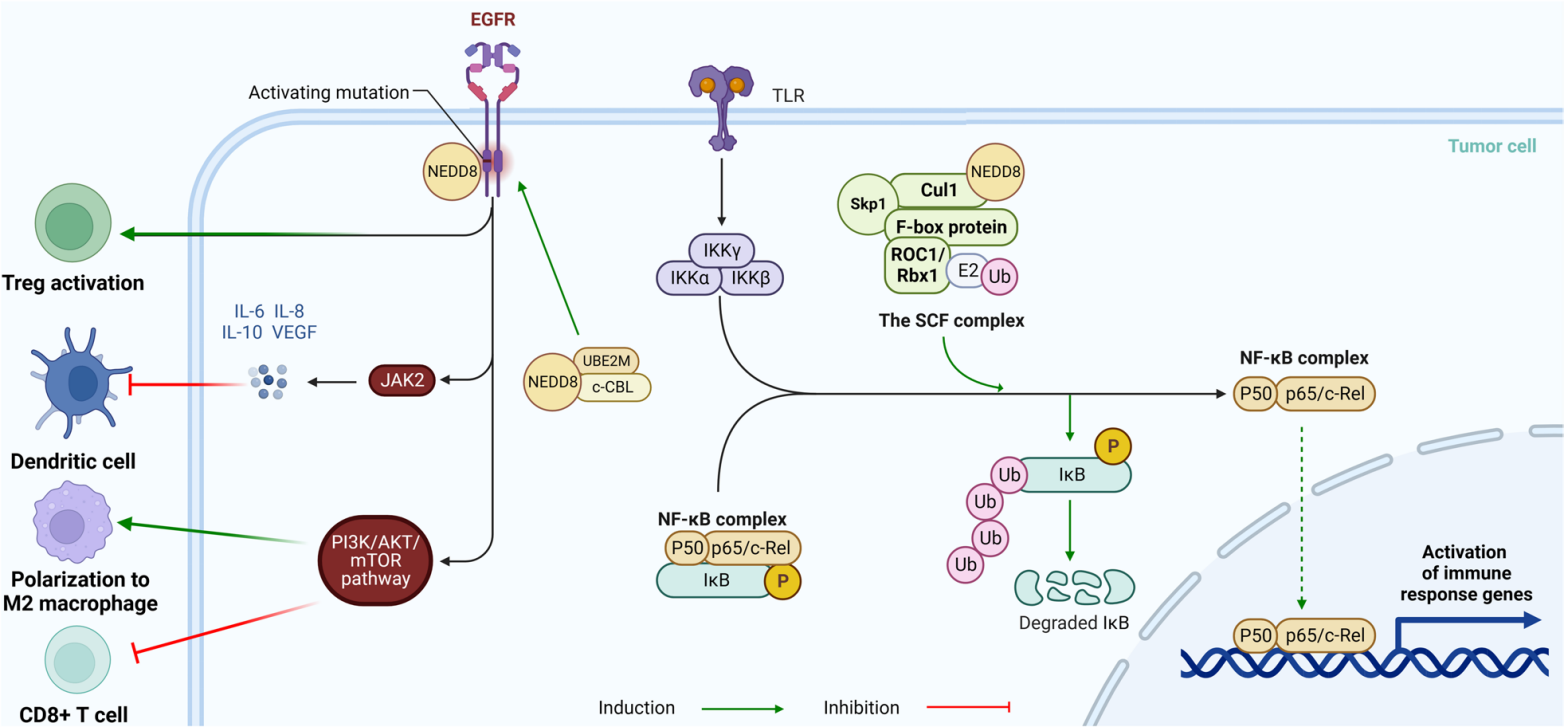
Neddylation plays a crucial role in the regulation of the NF-κB pathway and EGFR pathway, affecting several immune cells. In the NF-κB pathway, IκB inhibition and subsequent proteasomal degradation occur via IKK complex activation. The SCF complex, whose function is enhanced by neddylation, is instrumental in IκB ubiquitination. In the EGFR pathway, neddylation helps regulate the function of Tregs, dendritic cells, M2 macrophages, and CD8 + T cells. NF-κB, nuclear factor kappa-light-chain-enhancer of activated B cells; IκB, inhibitor of κB; IKK, IκB kinase; TLRs, Toll-like receptors; c-Rel, proto-oncogene c-Rel; SCF, Skp1-Cul1-F-box protein; Skp1, S-phase kinase-associated protein 1; Rbx1, ring-box 1; ROC1, regulator of Cullins 1; NEDD8, neural precursor cell expressed developmentally downregulated protein 8; UBE2M, ubiquitin-conjugating enzyme E2 M; c-CBL, casitas B-lineage lymphoma; Tregs, regulatory T cells. Created with BioRender.com

The NF-κB pathway plays a pivotal role in immune function. When danger signals are detected, innate immune cells activate NF-κB, promoting anti-tumor activity by releasing inflammatory cytokines. However, continuous activation can enhance tumor growth and survival [98]. In its inactive state, NF-κB is confined to the cytoplasm by the inhibitor protein IκB [99]. The recognition of pathogen-associated molecules like LPS, TNF, or IL-1 by toll-like receptors activates the IκB kinase (IKK) complex [100–102]. Once activated, IKK facilitates the degradation of IκB, freeing NF-κB to enter the nucleus [103] and stimulating the transcription of genes essential for immune responses and cell survival [104, 105]. The SCF complex, an integral E3 ubiquitin ligase in the NF-κB pathway, comprises four key components: Skp1, an adaptor molecule linking Cul1 and the F-box protein; Cul1, a scaffold protein that connects to Skp1 and the F-box protein at its N-terminal end and to ring-box 1 (Rbx1), also known as ROC1, at its C-terminal end; F-box proteins, responsible for guiding the SCF complex to its specific targets [106]; and Rbx1, which eases the ubiquitination of the target protein by attracting an E2 ubiquitin-conjugating enzyme to the complex. Neddylation, a process that involves the covalent attachment of NEDD8 to Cul1, amplifies the activity of the SCF complex [107, 108]. This enhancement allows the SCF complex to ubiquitinate its target proteins more effectively, such as the IκB protein [7]. The activated IKK complex can phosphorylate IκB to pIκB, enabling its recognition by the ubiquitin-binding enzyme SCF and degradation through ubiquitination and proteases. The neddylation process may boost SCF activity by activating Cul1 [108], thereby indirectly modulating the expression of the NF-κB pathway. Amplified neddylation boosts the polyubiquitination and proteasomal degradation of the IκB protein, directing the TME’s immune response toward cancer promotion (Fig. 3).

Similarly, EGFR significantly modulates immune cell behavior. Upon ligand binding, EGFR activation stimulates Treg activation, creating an immunosuppressive microenvironment that promotes tumor growth [109, 110]. It also promptes autophosphorylation, recruiting molecules like Janus kinase 2, which regulates the transcription of cytokine genes, including IL-6, IL-8, IL-10, and VEGF [73, 111–113]. These cytokines hinder dendritic cell maturation and functionality, thereby reducing their tumoricidal capacity [114]. Additionally, the EGFR/PI3K/AKT/mTOR pathway directs macrophage polarization towards the M2 phenotype, which secretes growth factors such as EGF, platelet-derived growth factor (PDGF), and transforming growth factor (TGF)-β, favoring tumor cell proliferation and survival [115, 116]. This pathway further diminishes the cytotoxicity of CD8 + T-cells, weakens the immune response against cancer cells, and promotes tumor progression [117–120]. The EGFR pathway is amplified by Casitas B-lineage lymphoma (c-Cbl), a ubiquitin ligase that modifies EGFR using the ubiquitin-like molecule NEDD8 [121]. This action triggers EGFR neddylation, leading to the endocytic internalization of EGFR and further augmentation of pathway expression [121]. In summary, neddylation of the EGFR indirectly promotes the occurrence and development of tumors by regulating the expression of various immune cells in the TME (Fig. 3).

#### Regulation of angiogenesis in TME by neddylation

Angiogenesis, a vital process in the TME, ensures nutrient and oxygen supply to tumors but often results in abnormal vasculature and hypoxia [57]. Such changes foster tumor malignancy and metastasis by augmenting vascular permeability [122, 123]. Although new vessels attract immune cells, their abnormalities can deter immune infiltration and assist tumor evasion [124]. Studies have shown that neddylation modulates the VEGF pathway by regulating the stability and degradation of VEGF receptors, thus affecting endothelial cell behavior crucial for new blood vessel formation [62]. Furthermore, neddylation stabilizes HIF-1α, a central player in the cellular response to hypoxia, ensuring the sustained activation of angiogenic genes such as VEGF [125]. Additionally, by targeting the enzymes responsible for ECM remodeling, neddylation influences ECM restructuring, a fundamental step in angiogenesis [126].

#### Regulatory mechanisms of neddylation in angiogenesis: insights from the hypoxia-inducible factor (HIF) pathways

**Fig. 4:**
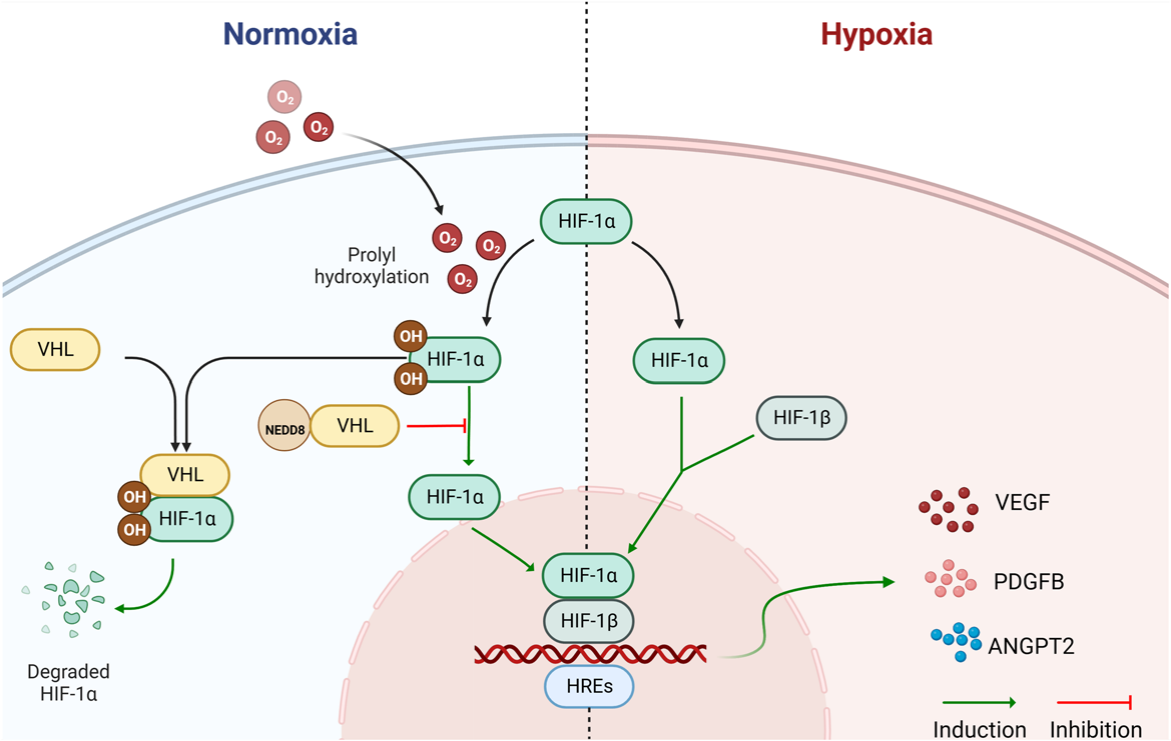
The HIF signaling pathway plays a vital role in tumor angiogenesis by adjusting HIF-1α levels based on oxygen availability, leading to angiogenesis-related gene activation under hypoxic conditions, while neddylation, by inhibiting HIF-1α degradation, can promote tumor growth and angiogenesis. HIF, hypoxia-inducible factor; VHL, von Hippel-Lindau; HREs, hypoxia response elements; VEGF, vascular endothelial growth factor; PDGFB, platelet-derived growth factor B; ANGPT2, angiopoietin 2; NEDD8, neural precursor cell expressed developmentally downregulated protein 8. Created with BioRender.com

The HIF pathway is pivotal for tumor angiogenesis. Primarily composed of HIF-1α and HIF-1β, HIF-1’s activity is oxygen-sensitive [127]. Under normal oxygen levels, HIF-1α is degraded due to hydroxylation by prolyl hydroxylase and is also restricted by factors inhibiting HIF-1 (FIH-1) [128]. In hypoxic TMEs, this degradation is halted, allowing HIF-1α to accumulate and pair with HIF-1β. This combined entity activates genes like VEGF, platelet-derived growth factor B (PDGFB), and angiopoietin 2, thereby promoting vessel formation, stability, and sprouting [129, 130]. Additionally, HIF-1 regulates genes that are essential for glucose metabolism and cell survival under hypoxic conditions [131]. Interestingly, von Hippel-lindau is a neddylation target. When neddylation occurs, it inhibits the subsequent degradation of HIF-1α, fostering its stabilization [132]. Such conditions may augment tumor malignancy. Therefore, maintaining the homeostasis of neddylation processes can enhance angiogenesis within the TME by regulating HIF-1α. This, in turn, profoundly impacts tumor development (Fig. 4).

#### Regulation of ECM and cancer-associated fibroblasts (CAFs) in TME by neddylation

The ECM and CAFs are essential for modulating tumor growth, metastasis, and therapeutic resistance. The ECM is a complex structural lattice composed of diverse biomolecules that serve not only as a physical scaffold but also as a mediator of tissue compartmentalization and intercellular signaling [133]. Alterations in the neddylation pathway within tumor cells can significantly reshape the communication dynamics between the tumor and its stroma, particularly influencing cytokine expression and release patterns and growth factors vital for stromal interactions [126]. Recent research highlights an upsurge in neddylation expression in tumor cell, intensifying tumor-stroma crosstalk and potentially hastening cancer progression [134, 135]. Conversely, reduced neddylation impedes pathways linked to fibroblast activation, specifically HMGA1 and HMGA2, angiogenesis markers like annexinA2 and agrin, and pivotal oncogenic routes such as NIK/NF-κB, TNF, Wnt, TGFβ, and mitogen-activated protein kinase [135]. Furthermore, there is an impact on ECM architecture, as neddylation can determine the activity of enzymes such as matrix metalloproteinases (MMPs) [126]. Altered neddylation of specific targets can lead to changes in the turnover of ECM components, affecting tissue stiffness, porosity, and the overall architecture [136, 137]. This modified ECM can directly promote malignancy by inducing mechanotransduction pathways [138], ECM degradation products can have bioactive properties that promote tumor growth and migration [139], and the ECM can control the availability of growth factors to tumor cells [140]. The above studies indicate that within the TME, the ECM is pivotal, and alterations in the neddylation pathway significantly influence tumor-stroma communication, ECM structural dynamics, and the mechanisms of tumor progression.

Similarly, in the TME, CAFs evolve from the transformation of typical fibroblasts and are instrumental in promoting tumor growth and invasion. As prevalent components of the tumor stroma, CAFs can stem from various sources [141] and secrete factors that stimulate cancer cell proliferation, enhance angiogenesis, modulate the immune response, and remodel the ECM [139]. CAFs also induce tumor-promoting inflammation, contribute to cancer cell metabolic reprogramming [142], and aid in therapeutic resistance, either by obstructing drug delivery or by producing factors that counteract drug-induced apoptosis [143]. Neddylation has emerged as a key regulator of CAFs in the TME [144]. This process potentially modulates CAF activation, altering their secretion profiles which influence cancer cell behaviors such as growth and invasiveness [145]. Additionally, it may influence CAF metabolic reprogramming, facilitating the metabolic needs of cancer cells and playing a role in the therapeutic resistance conferred by CAFs [135]. Furthermore, neddylation can shape CAF interactions with other TME cells, affecting overall cancer progression [144]. In summary, the neddylation pathway has emerged as a central modulator of CAF function, directing its interactions with neoplastic cells and subsequently affecting tumor behavior, offering a novel avenue for therapeutic interventions in oncology.

#### Regulatory mechanisms of neddylation in ECM and CAFs: insights from the TGF-β pathways

**Fig. 5:**
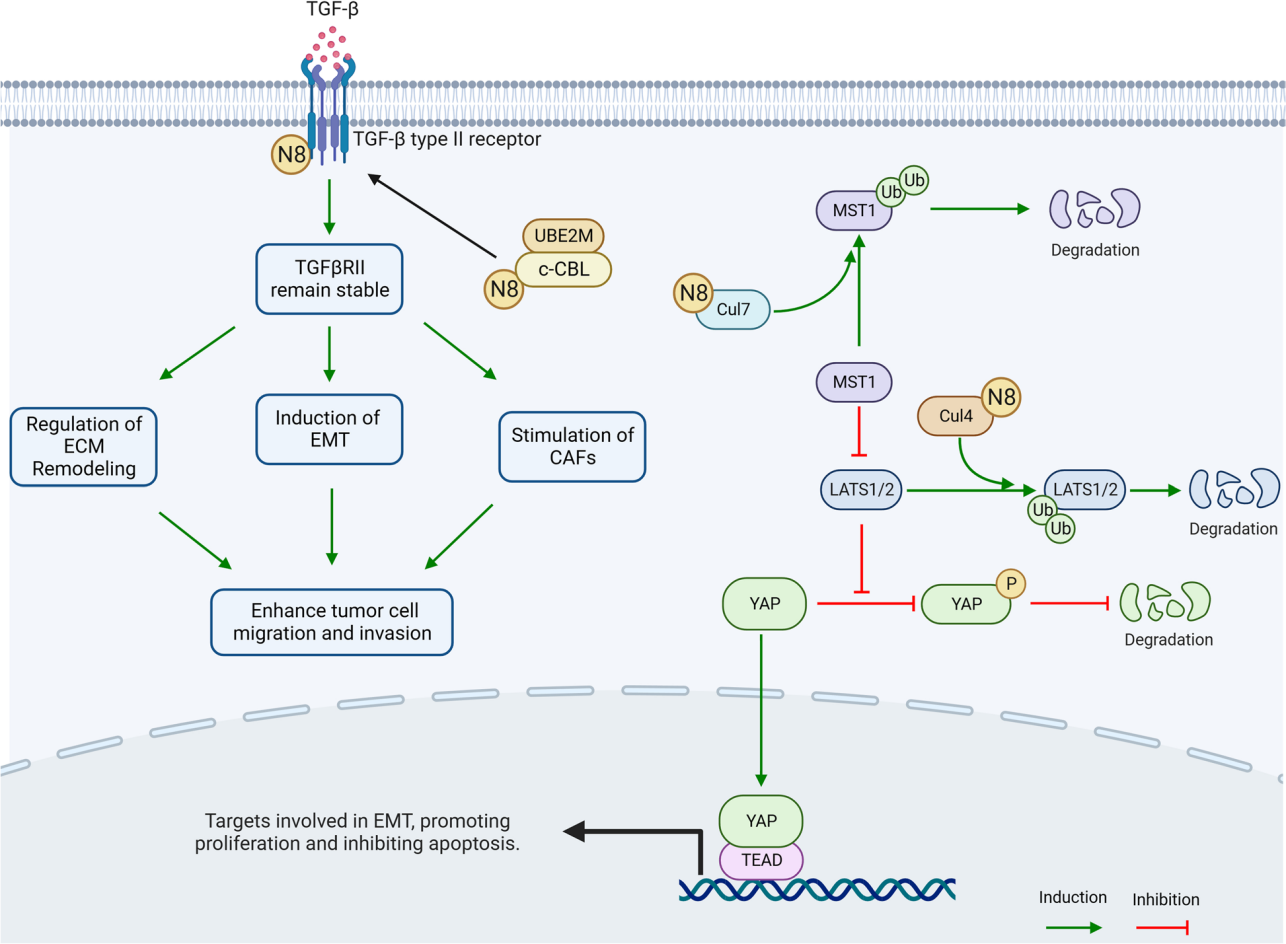
The TGF-β pathway, modulated by NEDD8, contributes to tumor progression by increasing ECM protein production, regulating ECM remodeling enzymes, promoting epithelial-to-mesenchymal transition, and activating CAFs. Furthermore, neddylation, facilitated by NEDD8, indirectly regulates the TGF-β pathway by stabilizing its signaling through c-CBL, potentially enhancing tumor invasiveness and malignancy. Simultaneously, the Hippo pathway, through the ubiquitination of MST1 and LATS1/2 by CUL7 and CUL4 respectively, plays a crucial role in tumorigenesis. MST1 inhibits the kinase cascade, including LATS1 and LATS2 activation, leading to the phosphorylation of the transcriptional co-activators YAP and TEAD, key downstream effectors of the Hippo pathway, thereby modulating tumor cell growth. Thus, both the TGF-β and Hippo pathways together form a complex network influencing tumor development. N8, neural precursor cell expressed developmentally downregulated protein 8; TGF-β, transforming growth factor-β; CAFs, cancer-associated fibroblasts; c-CBL, casitas b-lineage lymphoma; UBE2M, ubiquitin-conjugating enzyme E2 M; ECM, extracellular matrix; MST1, mammalian STE20-like protein kinase 1; LATS1 and LATS2, Large tumor suppressor kinase 1 and 2; YAP, Yes-associated protein; TEAD, TEA domain. Created with BioRender.com.

TGF-β significantly modulates the ECM within the TME. It stimulates the production of key ECM proteins, leading to denser ECM deposition and characteristic stromal rigidity in many solid tumors [146, 147]. Additionally, TGF-β regulates ECM-remodeling enzymes, influencing ECM integrity and promoting tumor cell invasion [148]. Through its promotion of EMT, TGF-β enhances epithelial cell motility and invasiveness, facilitating tumor progression [149]. In addition to its direct impact on the ECM, TGF-β indirectly influences ECM remodeling by activating CAFs. TGF-β, therefore, induces the differentiation of fibroblasts into myofibroblasts, a CAF subtype that produces substantial amounts of ECM components and ECM-remodeling enzymes [150]. A recent study demonstrated that the phosphorylation of TGFβ receptor 2 (TGFβRII) instigates the RING E3 ligase c-CBL activation, subsequently stabilizing and prolonging its signaling [151]. This mechanism targets TGFβRII for clathrin-mediated endocytosis under endogenous conditions, modulated by neddylation [151]. Thus, neddylation indirectly regulates the expression of the TGF-β pathway. Enhanced neddylation leads to amplified TGF-β pathway expression, increasing the invasiveness and migratory capabilities of the tumor, and consequently, fostering a higher degree of malignancy (Fig. 5).

#### Regulation of EMT in TME by neddylation

EMT significantly affects the TME, thereby advancing cancer progression, metastasis, and treatment resistance. Initiated by TME factors such as cytokines and hypoxia, EMT shifts cancer cells from epithelial to mesenchymal states, enhancing their invasiveness and resistance to apoptosis [152, 153]. This process aids in metastasis by promoting ECM degradation [154] and contributes to therapeutic resistance in various cancers, including lung cancer and melanoma [155–157], while also inducing cancer stem cell-like properties that intensify treatment challenges and recurrence [158]. Recent findings indicate that neddylation, pivotal for cancer cell migration via the PI3K-Akt pathway, when inhibited, upregulates HIF-1α, modulating EMT-activator ZEB1 in various cancer cell lines, underscoring its significant role in cancer progression and metastasis [159]. In breast cancer, neddylation modulates basal MKK7 activity, which affects the EMT phenotype [160]. Simultaneously, neddylation inhibitors (MLN4924) combined with celecoxib showed promising results in treating urothelial carcinoma, with celecoxib further enhancing the EMT-inhibitory effects of MLN4924 [161]. Thus, understanding the multifaceted role of neddylation in the EMT and its interactions with various drugs may pave the way for improved therapeutic strategies.

#### Regulatory mechanisms of neddylation in EMT: insights from the Hippo-YAP pathways

The Hippo- yes-associated protein (YAP) signaling pathway is a key regulator of EMT, orchestrating tissue homeostasis under normal conditions and driving tumor formation and progression when dysregulated. Typically, an active Hippo pathway phosphorylates YAP, sequesters and degrades them in the cytoplasm to suppress gene transcription, thereby promoting cell proliferation and inducing apoptosis [162]. Conversely, deregulation of the Hippo pathway triggers YAP dephosphorylation, causing nuclear translocation [163]. YAP forms complexes with TEA domains and other transcription factors, promoting the transcription of pro-proliferative and anti-apoptotic genes, thereby fostering tumor initiation, progression, and drug resistance [163]. This regulatory landscape is also shaped by neddylation, where the NEDD8 substrates CUL7 and CUL4, both ubiquitin ligases, promote the ubiquitination of mammalian STE20-like protein kinase 1 and potentially of large tumor suppressor kinase 1 and 2 (LATS1/2) [164, 165]. These events activate YAP signaling, suggesting that neddylation can amplify the transcription of genes that promote proliferation and inhibit apoptosis [166]. In addition to its role in tumorigenesis, YAP orchestrates cancer metastasis, guiding key processes such as tumor cell invasion and migration by remodeling the actin cytoskeleton and promoting EMT. YAP activates the transcription of genes governing cell motility, invasion, and EMT, such as connective tissue growth factors, fibroblast growth factors, and MMPs [167]. Their upregulation in circulating tumor cells, which are critical players in the metastatic process, underscores their crucial role in cancer spread [168] (Fig. 5).

### Targeting the neddylation pathway: emerging strategies in cancer therapeutics

#### Investigating deneddylating enzymes as potential therapeutics in oncology

Deneddylation, a counter-process to neddylation, is driven by the pivotal enzymes, CSN and SENP8 [169, 170]. Therapeutically, deneddylating enzymes are in the spotlight; inhibitors may have anti-cancer properties, while activators might be beneficial where enhanced deneddylation is required [171]. The CSN is a multifaceted multi-protein complex essential for cellular homeostasis [172]. Comprising eight distinct subunits, with CSN5 and CSN6 exhibiting isopeptidase activity that is pivotal for deneddylation, CSN plays a critical role in regulating the neddylation status of cullin proteins in CRLs [171, 173]. By modulating this status, CSN directly affects the ubiquitin-proteasome system, governing protein degradation, stability, and several of cellular processes such as cell cycle progression and DNA damage response [174]. Given the significance of CSNs in cellular processes, their targeting, especially in pathologies such as cancer, presents an intriguing therapeutic opportunity [171]. Emerging data suggest that CSN inhibitors, such as CSN5i-3 and the natural compound curcumin, operate predominantly by obstructing the CSN5’s deneddylase activity, leading to the hyper-neddylation of cullin proteins and consequent disruption of CRL function [171, 175]. Although these inhibitors have exhibited potential anti-cancer properties, challenges such as off-target effects, cellular redundancy, and potential toxicity underscore the need for meticulous research and optimization [49]. SENP8, also known as DEN1 or NEDP1, is a vital cysteine protease responsible for deneddylation of proteins [176]. By regulating the activity of CRLs, SENP8 ensures proper cellular homeostasis; however, its misregulation can lead to tumorigenesis by stabilizing pro-oncogenic proteins [176]. Consequently, SENP8 has emerged as a potential therapeutic target. Nonetheless, achieving specificity remains paramount, given the extensive array of neddylated proteins. In conclusion, although the roles of CSN and SENP8 in cellular dynamics are undeniable, translating them into mature, therapeutic solutions for tumors requires further refinement to mitigate potential risks.

#### Therapeutic effect of MLN4924: a current update

MLN4924, also known as pevonedistat, is a small-molecule inhibitor targeting NAE, a critical enzyme in the neddylation pathway [1]. Structurally, MLN4924, also known by its IUPAC name: (1 S,2 S,4R)-4-(4-((S)-2,3-dihydro-1 H-inden-1-ylamino)-7 H-pyrrolo[2,3-d]pyrimidin-7-yl)-2-hydroxycyclopentyl)methyl sulfamate, has a complex polycyclic arrangement. This includes a 7 H-pyrrolo[2,3-d]pyrimidinyl group linked to a cyclopentylmethyl sulfamate unit and an indenylaminyl substituent, which adds to its structural integrity and bioactivity [13].

Focusing on its mechanism of action, MLN4924 uniquely inhibits NAE, a key facilitator of the neddylation pathway that activates CRLs. MLN4924’s inhibition of NAE hinders the activation of CRLs, leading to the accumulation of CRL substrates and disruption of regular cellular processes [13]. This compound inhibits NAE by selectively forming a covalent NEDD8-MLN4924 adduct in situ that binds to NAE’s active site, thereby maintaining its activity [177]. This selectivity and the often dysregulated neddylation pathway in several cancers highlight MLN4924’s therapeutic potential [178].

This compound has shown promise in preclinical studies of numerous cancer types, including lymphoma, leukemia, and solid tumors, owing to its capacity to induce cell cycle arrest, apoptosis, senescence, and autophagy [179]. Its therapeutic efficacy and safety are currently being evaluated in clinical trials. Despite the potential of MLN4924 (pevonedistat) as a novel therapeutic for various cancer types in both preclinical and clinical studies, it is not without limitations. One such problem is drug resistance, which may arise from mutations in NAE1. These mutations alter the binding site of the drug, thereby dampening its inhibitory effects [180]. In addition, the increased expression of NEDD8-conjugated proteins has been observed in some MLN4924-resistant cancer cells [181]. Adverse side effects such as fatigue, nausea, vomiting, diarrhea, and anemia have also been reported in phase I trials [182]. Furthermore, while MLN4924 selectively inhibits NAE, it may not completely block all NEDD8 conjugation pathways, leaving other neddylation targets such as p53 and MDM2 unaffected [183]. Lastly, although MLN4924 has demonstrated efficacy in preclinical models, it may not always suffice as a standalone therapy. Certain cancer types may need to be treated in conjunction with other treatments to enhance their therapeutic efficacy [184].

#### Therapeutic effect of pevonedistat on patients with acute Myeloid Leukemia (AML) and Myelodysplastic Syndrome (MDS)

AML and MDS are both myeloid malignancies; however, they differ in their clinical presentations and challenges. MDS typically progresses slowly and does not require immediate therapeutic interventions [185]. By contrast, AML is characterized by a diverse clinical and molecular landscape, underscored by the uncontrolled proliferation of abnormally differentiated myeloid progenitor cells [186, 187]. Despite these differences, both MDS and AML are notorious for frequent relapses after chemotherapy and resistance to conventional treatments owing to the abnormal activation of various signaling pathways [188, 189]. These complications highlight the urgent need for innovative therapeutic strategies.

For a long time, the combined use of azacitidine and other chemotherapeutic drugs has had a certain effect, but owing to its non-negligible cytotoxicity, more complete drug combination therapy is warranted [190, 191]. Simultaneously, pevonedistat has emerged as a promising drug for treating acute myeloid leukaemia and myelodysplastic syndromes, demonstrating feasible administration and modest clinical efficacy in a phase 1 clinical trial (NCT00911066), despite hepatotoxicity and multi-organ failure identified as dose-limiting constraints [182]. Recent clinical trials (NCT03862157) have provided promising evidence for the role of pevonedistat in AML and MDS. When tested in combination with azacitidine and venetoclax in a phase 1/2 trial, the study targeted older adults recently diagnosed with secondary AML, MDS, or chronic myelomonocytic leukemia (CMML) and assessed their response rates to the specified drug combination [192]. Notably, an impressive complete remission or incomplete hematological recovery (CR/CRi) rate of 66% was observed in the AML cohort, whereas the MDS/CMML cohort demonstrated a robust overall response rate of 75%, signaling the potency of this drug combination [192]. However, the prevalence of common adverse events, such as infection and febrile neutropenia, must be considered [192]. Moreover, the research findings suggest potential molecular alterations that may contribute to the development of therapeutic resistance. As such, the ensuing implications from these results suggest that this innovative triplet combination could offer a beneficial treatment pathway for patients presenting with high-risk AML, MDS, or CMML.

In conclusion, an increasing number of clinical studies have begun to focus on combining pevonedistat and chemotherapeutic drugs such as azacitidine, offering a promising new direction for treating MDS and AML (Table 1).

#### Therapeutic effect of pevonedistat on patients with malignant Lymphoma

Despite advancements in treatment, the prognosis of patients with relapsed or refractory lymphoma remains poor. Although targeted therapies have cured some patients, managing refractory and relapsed conditions remains challenging [193]. Notably, although targeted therapeutic strategies have led to curative outcomes in a subset of patients with lymphoma, managing refractory and relapsed disease persistently presents a substantial challenge [194]. Therefore, pevonedistat has emerged as a promising therapeutic agent. Pevonedistat induces intrinsic apoptosis or senescence in diverse lymphoma cells in a cell line-dependent manner [195] and triggers G2 cell-cycle arrest in lymphoma cells, leading to apoptosis or senescence, while concurrently upregulating pro-apoptotic factors and downregulating anti-apoptotic factors [196]. It is also known for its inhibitory effect on NFκB activity, thereby re-sensitizing diffuse large B-cell lymphoma and primary chronic lymphocytic leukemia cells to extrinsic apoptosis [197]. Further evidence of the therapeutic potential of pevonedistat has been observed in preclinical lymphoma models. Particularly in activated B-cell diffuse large B-cell lymphoma cell lines, pevonedistat enhances the activity of various chemotherapeutic agents and inhibitors; when used in combination with ibrutinib or cytarabine, it improves survival rates in severe combined immunodeficiency mouse xenograft models [198].

Pevonedistat has shown promise not only in preclinical experiments but also in clinical trials. A phase I study investigating its effects in patients with relapsed or refractory myeloma and lymphoma demonstrated a well-tolerated profile with minimal myelosuppression and no treatment-related deaths [184]. These outcomes suggest that pevonedistat could potentially be effective in managing refractory lymphoma, as evidenced by some patients achieving disease stability or partial response. In conclusion, this drug has shown encouraging results in clinical trials, both as a standalone treatment and in conjunction with other chemotherapies or targeted therapy regimens (Table 1).

#### Therapeutic effect of pevonedistat on patients with solid tumors

Primarily effective against various solid tumors such as those of the colon and lung as exhibited in preclinical studies, pevonedistat’s potent anti-tumor activity extends across multiple tumor types [199–203], and it has been proven efficacious in tumor xenograft mouse models, where it curtails tumor growth through the inhibition of NEDD8 conjugation and increasing NAE inhibition following both single and repeated doses [204]. As with hematological tumors, pevonedistat continues to be used in conjunction with chemotherapeutic drugs to treat solid tumors with significant success (Table 1). An earlier study revealed that pevonedistat, under phase I/II clinical trials as a potential glioblastoma treatment, was found that when combined with anti-PD-L1 therapy, the therapeutic efficacy significantly improves in vivo by effectively restoring T cell sensitivity [205]. These findings provide investigators with increased confidence in potentially combining pevonedistat with targeted therapies.

The latest Clinical Trial (NCT03486314) demonstrated that MLN4924 not only inhibited cell viability and induced apoptosis in human umbilical vascular endothelial cells but also disrupted cell cycle checkpoint regulators suppressed angiogenic activity and cell migration, decreased UBC12 levels (indicating VEGF-activated neddylation pathway involvement), and inhibiteds tumor growth in mouse models of four different types of cancer [206]. Given the significant anti-tumor effects observed in various solid tumors with the neddylation inhibitor, pevonedistat can be used in combination treatments for advanced solid tumors, potentially paving the way for new therapeutic strategies.

**Table 1:** Clinical trials of MLN4924 on AML and MDS, malignant lymphoma, and solid tumors. Information retrieved from ClinicalTrials.gov

Cancer type	Drug name	Combination therapy	Trial name	Phase	Condition or disease	Primary Outcome Measures	Enrollment	Sponsors and Collaborators	Actual Study Start Date	Actual/Estimated Study Completion Date	Recruitment Status	Study identifier
AML and MDS	Pevonedistat	Venetoclax, Azacitidine	A Study of Pevonedistat and Venetoclax Combined with Azacitidine to Treat AML in Adults Unable to Receive Intensive Chemotherapy	Phase II	Adult patients with AML who are unable to be treated with intensive chemotherapy.	EFS	164 participants	Takeda	October 13, 2020	September 6, 2022	Active, not recruiting	NCT04266795
	Pevonedistat	Cytarabine	Pevonedistat and Low Dose Cytarabine in Adult Patients with AML and MDS	Phase I	Adult patients with Relapsed/Refractory AML and Advanced MDS	Safety Profile, MTD, RP2D	12 participants	Justin Watts, MD; Takeda	May 21, 2018	June 25, 2021	Completed	NCT03459859
	Pevonedistat	Azacitidine	Study of MLN4924 Plus Azacitidine in Treatment-naive Participants with AML Who Are 60 Years or Older	Phase I	Treatment-Naïve Patients with Acute Myelogenous Leukemia Who Are 60 Years or Older	TEAEs, SAEs	64 participants	Millennium Pharmaceuticals, Inc.	April 10, 2013	April 8, 2018	Completed	NCT01814826
	Pevonedistat	Azacitidine, Venetoclax	Pevonedistat, Azacitidine (or Decitabine), and Venetoclax for the Treatment of Patients With AML	Phase I	Patients With AML	RP2D, Toxicity profile	24 participants	Medical College of Wisconsin	January 13, 2020	December 1, 2025	Active, not recruiting	NCT04172844
	Pevonedistat	Azacitidine	Treatment of MDS/AML Patients with an Impending Hematological Relapse with AZA or ATA and Pevonedistat	Phase II	MDS/AML Patients with an Impending Hematological Relapse	MRD	14 participants	University of Leipzig, Millennium Pharmaceuticals, Inc.	January 1, 2021	January 31, 2023	Completed	NCT04712942
	Pevonedistat	Cytarabine, Idarubicin	Pevonedistat, Cytarabine, and Idarubicin in Treating Patients With AML	Phase I/II	Patients with acute myeloid leukemia	Composite complete response rate; Incidence of adverse events	53 participants	University of Southern California; NCI	April 18, 2018	October 13, 2025	Active, not recruiting	NCT03330821
	Pevonedistat	Belinostat	Pevonedistat and Belinostat in Treating Patients with Relapsed or Refractory AML or MDS	Phase I	Patients with Relapsed/Refractory AML or MDS.	RP2D	30 participants	NCI	June 20, 2019	July 1, 2024	Active, not recruiting	NCT03772925
	Pevonedistat	Azacitidine, Cytarabine, Fludarabine Phosphate, Methotrexate, Therapeutic Hydrocortisone	Pevonedistat, Azacitidine, Fludarabine Phosphate, and Cytarabine in Treating Patients with Relapsed or Refractory AML or MDS	Phase I	Patients with Relapsed/Refractory AML or MDS.	Dose Limiting Toxicities; Adverse Events	12 participants	NCI; Children’s Oncology Group	May 1, 2019	October 5, 2023	Active, not recruiting	NCT03813147
	Pevonedistat	Decitabine	Pevonedistat and Decitabine in Treating Patients with High -Risk AML	Phase I	Patients with high-risk AML.	Adverse Events; DLT; MTD	30 participants	City of Hope Medical Center; NCI	August 21, 2017	December 15, 2023	Active, not recruiting	NCT03009240
	Pevonedistat	Azacitidine	Pevonedistat Plus Azacitidine Versus Single-Agent Azacitidine as First-Line Treatment for Participants with Higher-Risk HR MDS, CMML, or Low-Blast AML	Phase III	Patients With HR MDS, CMML, or Low-Blast AML	EFS	454 participants	Takeda; Takeda Development Center Americas, Inc.	November 28, 2017	June 30, 2023	Active, not recruiting	NCT03268954
	Pevonedistat	Azacitidine	An Efficacy and Safety Study of Pevonedistat Plus Azacitidine Versus Single-Agent Azacitidine in Participants with HR MDS, CMML and Low-Blast AML	Phase II	Participants with HR-MDS or CMML, or low-blast AML	OS	120 participants	Millennium Pharmaceuticals, Inc.	April 14, 2016	July 23, 2021	Completed	NCT02610777
	Pevonedistat	Azacitidine	Study to Compare Azacitidine Plus Pevonedistat Versus Azacitidine in Patients with AML Not Eligible for Standard Chemotherapy	Phase III	Patients with newly diagnosed AML not eligible for intensive chemotherapy	OS	302 participants	PETHEMA Foundation; Millennium Pharmaceuticals, Inc.; Dynamic Science S.L.	September 24, 2019	June 30, 2023	Active, not recruiting	NCT04090736
Malignant lymphoma	MLN4924	None	Study of MLN4924, a Novel Inhibitor of Nedd8 Activating Enzyme, in Adult Patients with Lymphoma or Multiple Myeloma	Phase I	Adult patients with lymphoma or multiple myeloma.	Safety and tolerability	56 participants	Millennium Pharmaceuticals, Inc.	July 25, 2008	November 18, 2013	Completed	NCT00722488
	Pevonedistat	Ibrutinib	Pevonedistat and Ibrutinib in Treating Participants with Relapsed or Refractory CLL or NHL	Phase I	Participants with chronic lymphocytic leukemia or NHL that has come back or has stopped responding to other treatments.	DLTs, AEs, SAEs	18 participants	City of Hope Medical Center; National Cancer Institute (NCI)	March 22, 2018	December 2, 2023	Active, not recruiting	NCT03479268
	Pevonedistat	Irinotecan, Temozolomide	Pevonedistat, Irinotecan, and Temozolomide in Treating Patients with Recurrent or Refractory Solid Tumors or Lymphoma	Phase I	Relapsed/Refractory Solid Tumors or Lymphoma.	MTD, RP2D	30 participants	Children’s Oncology Group; National Cancer Institute (NCI)	November 13, 2017	September 30, 2023	Active, not recruiting	NCT03323034
	Pevonedistat	Vincristine, Dexamethasone, PEG-asparaginase, Doxorubicin, Cytarabine, Methotrexate, Hydrocortisone	Pevonedistat with VXLD Chemotherapy for Adolescent/Young Adults with Relapsed/Refractory ALL or Lymphoblastic NHL	Phase I	Adolescent/Young Adults with Relapsed/Refractory ALL or Lymphoblastic NHL.	Toxicity, MTD	6 participants	Julio Barredo, MD; Takeda	March 25, 2019	October 12, 2022	Completed	NCT03349281
	MLN4924	Azacitidine	MLN4924 for the Treatment of AML, MDS, and ALL	Phase I	AML, MDS, and ALL	Adverse events, serious adverse events, assessments of clinical laboratory values, and vital sign measurements	72 participants	Millennium Pharmaceuticals, Inc.	June 1, 2009	December 5, 2013	Completed	NCT00911066
Solid Tumors	MLN4924	Paclitaxel, Gemcitabine, Docetaxel, Carboplatin	Dose Escalation, Multi-arm Study of MLN4924 Plus Docetaxel, Gemcitabine, or Combination of Carboplatin and Paclitaxel in Participants with Solid Tumors	Phase I	Solid tumors.	TEAEs, SAEs	64 participants	Millennium Pharmaceuticals, Inc.	June 10, 2013	May 21, 2018	Completed	NCT01862328
	MLN4924	Fluconazole, Itraconazole, Docetaxel, Carboplatin, Paclitaxel	Effects of Fluconazole and Itraconazole CYP3A-Mediated Inhibition on the Pharmacokinetics, Safety, and Tolerability of MLN4924 in Participants with Advanced Solid Tumors	Phase I	Solid tumors.	Area Under the Plasma Concentration-time Curve	51 participants	Millennium Pharmaceuticals, Inc.	April 1, 2014	June 5, 2017	Completed	NCT02122770
	Pevonedistat	Irinotecan, Temozolomide	Pevonedistat, Irinotecan, and Temozolomide in Treating Patients with Recurrent or Refractory Solid Tumors or Lymphoma	Phase I	Relapsed/Refractory Solid Tumors or Lymphoma	MTD, RP2D	30 participants	Children’s Oncology Group, NCI	November 13, 2017	September 30, 2023	Active, not recruiting	NCT03323034
	Pevonedistat	Rifampin, Docetaxel, Carboplatin, Paclitaxel	A Study to Evaluate the Effects of Rifampin on Pharmacokinetics (PK) of Pevonedistat in Participants with Advanced Solid Tumors	Phase I	Advanced Solid Tumors	Cmax, Area Under the Plasma Concentration-time Curve	20 participants	Millennium Pharmaceuticals, Inc.	August 13, 2018	February 28, 2021	Completed	NCT03486314
	Pevonedistat	Azacitidine, Docetaxel, Paclitaxel, Carboplatin	A Study of Pevonedistat in People with Blood Cancers or Solid Tumors with Kidney or Liver Problems	Phase I	Blood cancers or solid tumors	Area Under the Plasma Concentration-time Curve	17 participants	Takeda	July 10, 2019	April 19, 2022	Completed	NCT03814005
	Pevonedistat	Docetaxel, Carboplatin, Paclitaxel	A Study to Evaluate the Effects of Pevonedistat on the Corrected QT (QTc) Interval in Participants with Advanced Solid Tumors	Phase I	Advanced Solid Tumors	QTcF	68 participants	Millennium Pharmaceuticals, Inc.	November 6, 2017	March 28, 2023	Completed	NCT03330106
	Pevonedistat	Carboplatin, Paclitaxel	Testing the Combination of MLN4924 (Pevonedistat), Carboplatin, and Paclitaxel in Patients with Advanced NSCLC Who Have Previously Been Treated with Immunotherapy	Phase II	Advanced NSCLC Who Have Previously Been Treated with Immunotherapy	ORR	24 participants	NCI	September 3, 2019	March 9, 2024	Active, not recruiting	NCT03965689
	Pevonedistat	Carboplatin, Paclitaxel	Testing the Combination of Pevonedistat with Chemotherapy for Bile Duct Cancer of the Liver	Phase II	Bile Duct Cancer of the Liver	Objective response rate	52 participants	NCI	January 31, 2020	October 1, 2023	Active, not recruiting	NCT04175912

*AML *Acute myeloid leukemia, *MDS *Myelodysplastic syndromes, *HR MDS *Higher-risk myelodysplastic syndromes, *CMML* Chronic myelomonocytic leukemia, *EFS* Event-free survival, *MTD *maximum tolerated dose, *TEAEs* Treatment-emergent adverse events, *SAEs *Serious adverse events, *RP2D* Recommended phase 2 dose, *MRD* measurable residual disease, *DLTs* Dose-limiting toxicities, *OS* Overall survival, *NCI *National Cancer Institute, *CLL* Chronic lymphocytic leukemia, *NHL* Non-Hodgkin lymphoma, *ALL* Acute lymphoblastic leukemia, *AEs *Adverse events, *Cmax *Maximum observed plasma concentration, *QTcF *Fridericia-corrected QT interval, *ORR *Overall response rate, *NSCLC *Non-small cell lung cancer

## Conclusions

In oncological research, there has been a marked transition from an exclusive concentration on malignant cells to a comprehensive exploration of the TME, encompassing the interplay of malignant cells, immune cells, stromal cells, the ECM, and the molecular constituents that interface with the tumor [207–209]. The TME plays an instrumental role in tumorigenesis, metastasis, and the response to therapy, making it a rich source of potential therapeutic targets. However, future studies must address several challeng to harness this potential fully. Both intra- and inter-tumoral, heterogeneity pose significant therapeutic resistance challenges [210]. Intra-tumoral variations encompass genetic, epigenetic, and phenotypic disparities within a tumor, which are influenced by factors such as distinct cancer cell evolutionary paths and microenvironmental variations [211, 212]. Inter-tumor differences arise from variances in genetics, environment, and immune responses among tumors, even within the same patient [213], and can foster the development of resistant clones, complicating treatment outcomes [214]. To address the challenges posed by tumor heterogeneity, it is essential to understand the underlying signaling mechanisms within the TME and identify potential therapeutic targets. Emphasizing the need for in-depth mechanistic studies, our research reveals the extensive involvement of neddylation in the TME’s regulatory processes, encompassing pathways like p53, PI3K/AKT/mTOR, NF-κB, EGFR, HIF, TGF-β, and Hippo-YAP. The centrality of neddylation in tumor progression underscores its potential as a novel therapeutic intervention.

Metastasis, a primary cause of cancer-related fatalities, depends on the formation of pre-metastatic niches. These niches, which are altered regions within potential metastatic sites, are primed by the primary tumor to facilitate metastatic cell growth. Tumors achieve this through factor secretion, cell recruitment [59], induction of inflammation, and modification of vascular and extracellular structures [215, 216]. Although progress has been made in understanding these niches, the exact mechanisms and pathways involved, especially those related to the EMT within the TME, remain elusive [168]. Through an in-depth analysis, we determined that neddylation critically influences the formation of pre-metastatic niches. It directly governs cancer cell migration, invasion, and interactions within the TME, particularly by modulating cytokine secretion and growth factors [144]. Neddylation also plays a crucial role in signaling pathways related to metastasis and has noteworthy implications for immune evasion [5]. Its significant impact on ECM remodeling, essential for fostering a favorable environment for metastatic cells, further underscores the potential of neddylation as a therapeutic target [135].

Recently, Mln4924 emerged as a major breakthrough. By directly inhibiting neddylation modifications within cell pathways, MLN4924 disrupts many signaling pathways that could potentially interfere with the TME and promote tumor progression [217]. The efficacy of MLN4924, particularly when used in conjunction with chemotherapy, has been demonstrated in numerous clinical trials. These insights may aid in the development of more effective cancer treatment strategies. In summary, our findings highlight the profound impact of neddylation on TME, offering promising avenues for enhanced cancer treatment strategies.

## Data Availability

The datasets supporting the conclusions are included within this article.

## Glossary

AEs: Adverse events.
ALL: Acute lymphoblastic leukemia.
AML: Acute myeloid leukemia.
ANGPT2: Angiopoietin 2.
BCA3: Breast cancer-associated protein 3.
CAFs: Cancer-associated fibroblasts.
c-CBL: Casitas b-lineage lymphoma.
CLL: Chronic lymphocytic leukemia.
CMML: Chronic myelomonocytic leukemia.
CR: Complete remission.
CRi: Complete remission with incomplete blood count recovery.
CRLs: Cullin-RING ligases.
CUL: Cullin.
CSN5: COP9 signalosome subunit 5.
DCs: Dendritic cells.
DCAF: DDB1- and CUL4-Associated Factor.
DDB1: DNA damage-binding protein 1.
DLTs: Incidence of dose-limiting toxicities.
ECM: Extracellular matrix.
EFS: Event-free survival.
EMT: Epithelial-to-mesenchymal transition.
FIH: Factor inhibiting HIF.
GPCRs: G protein-coupled receptors.
HIF: Hypoxia-inducible factor.
HR MDS: Higher-risk myelodysplastic syndromes.
HREs: Hypoxia response elements.
IKK: IκB kinase.
IL: Interleukin.
JAB1: Jun activation domain-binding brotein 1.
JAMM: JAB1/MPN/Mov34 metalloenzyme.
LATS1/2: Large tumor suppressor kinase 1 and 2.
LPS: Lipopolysaccharides.
MDM2: Mouse double minute 2 homolog.
MDS: Myelodysplastic syndromes.
MMPs: Matrix metalloproteinases.
Mov34: Modifier of variegation 3–4.
MPN: Mpr1/Pad1 N-terminal domain.
Mpr1: Multidrug resistance-associated protein 1.
MRD: Measurable residual disease.
MST1: Mammalian STE20-like protein kinase 1.
MTD: Maximum tolerated dose.
mTORC1: mTOR complex 1.
NAE: NEDD8 activating enzyme.
NCI: National cancer institute.
NEDD8: Neural precursor cell expressed developmentally downregulated protein 8.
NEDP1: NEDD8 protease 1.
NF-κB: Nuclear factor kappa-light-chain-enhancer of activated B cells.
NHL: Non-Hodgkin lymphoma.
NSCLC: Non-small cell lung cancer.
OS: Overall survival.
Pad1: Phenylacrylic acid decarboxylase 1.
PARC: p53-associated parkin-like cytoplasmic protein.
PDGFB: Platelet-derived growth factor B.
PDK1: 3-Phosphoinositide Dependent Protein Kinase-1.
PD-L1: Programmed death-ligand 1.
PHD: Prolyl hydroxylase domain.
PI3K: Phosphoinositide 3-kinases.
PIP2: Phosphatidylinositol 4,5-bisphosphate.
PIP3: Phosphatidylinositol 3,4,5-trisphosphate.
RBX: RING box protein.
Rheb: Ras homolog enriched in brain.
ROC: Regulator of Cullins.
RP2D: Recommended phase 2 dose.
RPL11: Ribosomal protein L11.
RTKs: Receptor tyrosine kinases.
SAEs: Serious adverse events.
SAG: Sensitive to apoptosis gene.
SCF: Skp1-Cul1-F-box protein.
SENP8: Sentrin/SUMO-specific protease 8.
Skp1: S-phase kinase-associated protein 1.
TAMs: Tumor-associated macrophages.
TEAD: TEA domain.
TEAEs: Treatment-emergent adverse events.
TGFβ: Transforming growth factor-β.
TME: Tumor microenvironment.
TNF: Tumor necrosis factor.
Tregs: Regulatory T cells.
TSC: Tuberous sclerosis complex.
UBA3: Ubiquitin-like modifier activating enzyme 3.
UBC12: Ubiquitin conjugating enzyme E2 M.
UBE2F: Ubiquitin-conjugating enzyme E2 F.
UBE2M: Ubiquitin-conjugating enzyme E2 M.
UBL: Ubiquitin-like.
UCH-L3: Ubiquitin C-terminal hydrolase-L3.
UPS: Ubiquitin-proteasome system.
VEGF: Vascular endothelial growth factor.
VHL: Von hippel-lindau.
